# EMAC, Retromer, and VSRs: do they connect?

**DOI:** 10.1007/s00709-020-01543-8

**Published:** 2020-08-11

**Authors:** Rumen Ivanov, David G. Robinson

**Affiliations:** 1 Institute of Botany, Heinrich Heine University, 40225 Düsseldorf, Germany; 2 Centre for Organismal Studies, University of Heidelberg, 69117 Heidelberg, Germany

**Keywords:** Endomembrane trafficking, Retromer, VPS26-VPS35-VPS29, Sorting nexin, SNX, Subcellular localization

## Abstract

Eukaryotic organisms share many common features in terms of endomembrane trafficking. This fact has helped plant scientists to propose testable hypotheses on how plant intracellular membrane trafficking is achieved and regulated based on knowledge from yeast and mammals. However, when a new compartment has been identified in a plant cell that has a vesicle tethering complex located at a position which is completely different to its counterpart in yeast and mammalian cells, caution is demanded when interpreting possible interactions with other trafficking elements. This is exemplified by the recently discovered EMAC (ER and microtubule-associated compartment). It has been postulated that this compartment is the recipient of vacuolar sorting receptors (VSRs) transported retrogradely via “retromer vesicles” from a post-Golgi location. Unfortunately, this suggestion was based entirely on our knowledge of retromer from yeast and mammalian cells, and did not take into account the available literature on the composition, localization, and function of the plant retromer. It also lacked reference to recent contradictory findings on VSR trafficking. In this short article, we have tried to rectify this situation, pointing out that plant retromer may not function as a pentameric complex of two subunits: the retromer core and the sorting nexins.

Exchange of material between intracellular membrane compartments is mediated by vesicles and is essential for the function of eukaryotic organisms. Endomembrane vesicular transport is orchestrated by regulatory protein complexes, whose subunits are often evolutionary conserved between distantly related organisms (Bethune and Wieland 2018; Brandizzi 2018; Heucken and Ivanov 2018). However, evidence shows that protein complex composition and subunit function might significantly differ between species, making inference of functions a tricky task.

In a recently reported elegant screen for plant vacuolar trafficking regulators, Delgadillo et al. (2020) identified a number of proteins, including vacuolar sorting receptors (VSRs) and the Arabidopsis VPS51 and VPS54 homologs. Since VSP51 and VPS54 are components of the GARP tethering complex, transient expression studies were performed on tobacco epidermal cells with RFP-VPS51 and VPS54-GFP to elucidate the nature and localization of the plant GARP complex. In yeast and mammals, the GARP complex locates strictly to the *trans*-Golgi network (TGN) where it serves to tether incoming endosome-derived retrograde transport vesicles. Thus, it came as a surprise to discover that neither fluorescently tagged VPS51 nor VPS54 colcalized with standard TGN, Golgi, or multivesicular body (MVB) markers. Furthermore, whereas treatment with the Golgi-vesiculation inhibitor brefeldin A (BFA) typically leads to a redistribution of the Golgi membrane marker YFP-MEMB12 into the ER, the VPS51 and VPS54 signals remained unaffected. This was also the case after treatment with wortmannin which causes MVBs to enlarge via homotypic fusion (Wang et al. 2009). Interestingly, the RFP-VPS51 signals were visualized in a typical “beads-on-a string” pattern, which closely followed the orientation of ER tubules, and with microtubules labelled with GFP-MAP4. Consequently, the authors termed the GARP-positive structures EMAC, a previously unidentified ER- and microtubule-associated compartment. Intriguingly, EMACs were relatively immobile and their organization was not affected by microtubule depolymerisation, which led the authors to propose they might act as microtubule anchors. As VPS51 was found to interact with kinesin-type microtubule motors, an analogy was made with the previously described SORTING NEXIN1 (SNX1)–dependent protein recycling, which relies on an interaction between SNX1 and the microtubule plus end-associated protein CLASP (Ambrose et al. 2013).

While the data presented in the paper appears solid and highly novel, we find that the discussion surprisingly ignores current developments in some aspects of plant endomembrane trafficking. Delgadillo et al. (2020) postulate that VSR recycling and the SNX1-microtubule connection are linked to the function of the retromer complex. Considering that experiments on plant retromer were not presented in this paper, this is somewhat disturbing. In addition, the authors make extensive use of the term “retromer vesicles,” which is only occasionally used in the mammalian literature and never in plants, all for a good reason. Their conclusions are based on reference to yeast and mammalian systems, but given the fact that a lot of data is already available on plant retromer homologs and that this complex seems to function quite differently in plants, we regard the discussion section of Delgadillo et al. (2020) as not being truly representative of the literature at hand. It is also an example that one must be careful in extrapolating data from yeast and mammalian systems, especially when the location of the protein complex in question (the GARP complex) is different in plant cells.

Retromer is a trimeric protein encoded by three vacuolar protein sorting genes (*vps35*, *vps29*, and *vps26*) which are expressed in all eukaryotes. In yeast and mammalian cells, a second complex, usually containing two sorting nexins (SNX), is associated with the trimeric core retromer complex. Whereas Vps35p binds to endosomal membranes and was considered to interact with the cytosolic domain of sorting receptors for acid hydrolases (e.g., cation-independent mannosyl 6-phosphate receptor, CI-MPR, in mammals, vps10p in yeast), the SNXs, having a BAR-domain, were believed to be responsible for inducing membrane curvature and therefore driving tubule formation at the endosomal membrane (Carlton et al. 2004). As originally perceived, the retromer complex plays a key role in receptor recycling to the TGN. With the identification of retromer core homologs in plants, their localization to the MVB, and immunological evidence for an interaction between VPS35 and a vacuolar sorting receptor (VSR_At-1_) (Oliviusson et al. 2006), and after the demonstration that plants also have functional SNXs (Jaillais et al. 2006), it therefore became understandable for plant scientists to assume that VSR recycling from the MVB in plants operated in a similar manner as for the mannosyl 6-phosphate receptor (MPR) in mammals (Fig. 1a). This analogy is, however, not upheld by more recent research.

**Fig. 1:**
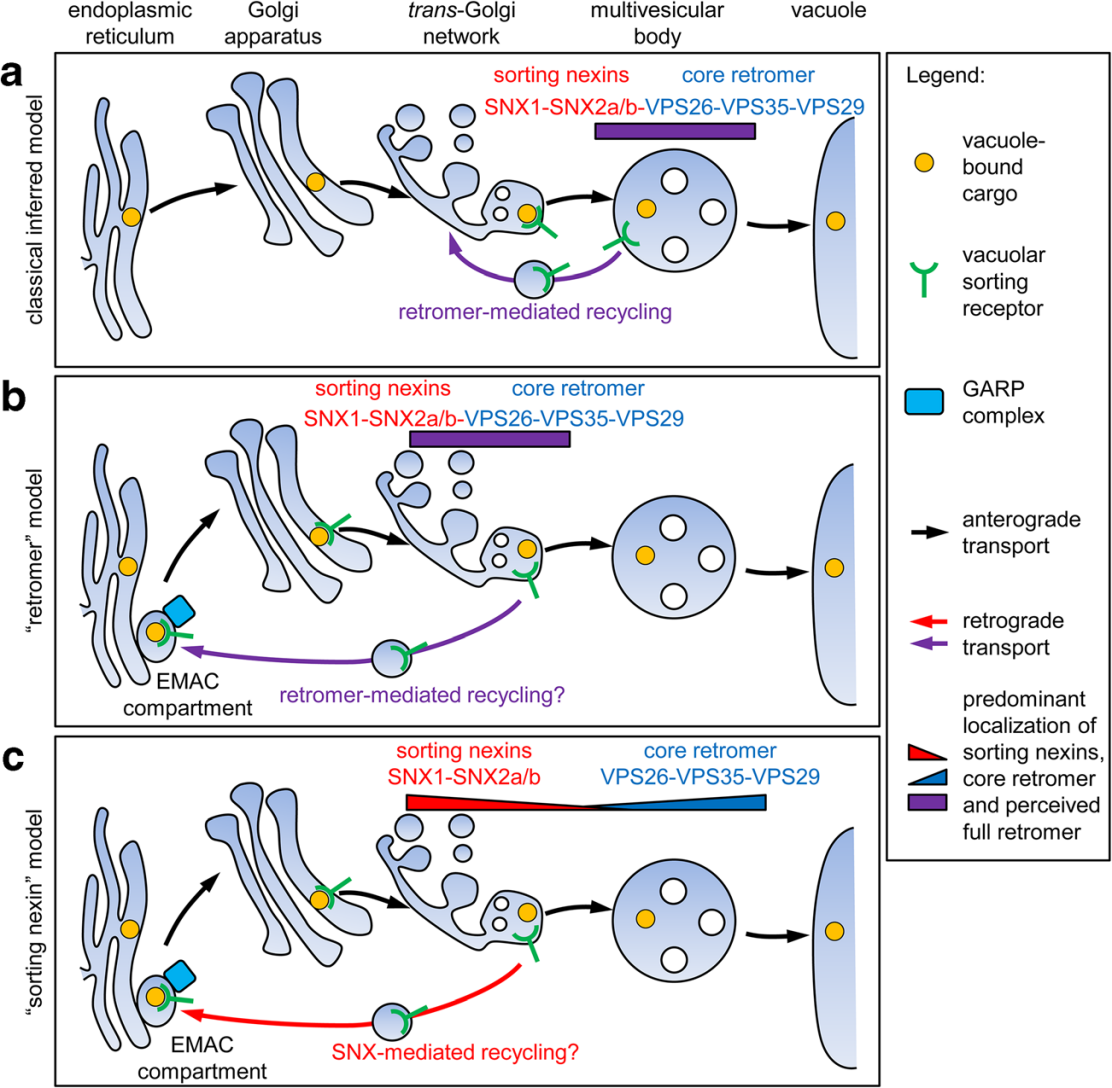
Three views of the role of retromer and sorting nexins in plant vacuolar sorting receptor trafficking. (a) The classical model inferred from the role of the yeast retromer. In this view, retromer as a pentameric complex is involved in retrograde trafficking of sorting receptors between the MVB and the TGN. (b) A new model based on recent data of vacuolar sorting receptor (VSR) recycling from the TGN. It includes the newly reported EMAC compartment as a potential acceptor of recycled VSRs and place of interaction between VSRs and vacuole-bound cargo. The model is based on Scheuring and Kleine-Vehn (2020). The models in A and B assume the existence in plants of a yeast-like retromer complex consisting of a sorting nexin dimer bound to a VPS26-VPS35-VPS29 trimer. (c) A model that takes into account the fact that SNX1-SNX2a/b complex and the core retromer are spatially separate, do not interact and function at different stages of the endomembrane system. VSR recycling is therefore likely to depend on sorting nexin function. The core retromer components VPS26, VPS29, and VPS35 are likely not involved in this process, neither as a trimer nor in the form of a full retromer complex, as known from yeast and mammalian systems

Although a physical interaction between the retromer core complex and the SNXs is well-established for yeast and mammalian cells, there is no solid evidence, biochemical or functional, that the VPS35-VPS29-VPS26 trimer and the SNXs function in the same complex in plants. Indeed, the fact that AtVPS29 correctly associates with membranes in SNX-BAR loss of function plants strongly suggests that a SNX subcomplex is not required for recruitment of the retromer core (Pourcher et al. 2010). Moreover, whereas *vps35b*, *vps35c* double, and *vps35a*, *vps35b*, *vps35c* triple mutants display major developmental defects including abnormal trafficking of storage proteins (Yamazaki et al. 2008), a *snx1*, *snx2a*, *snx2b* triple mutant exhibits only minor defects (Pourcher et al. 2010).

Based on the direct immunogold detection of VPS35 on mature MVBs (Oliviusson et al. 2006) and the observation that stably expressed core retromer subunits localize to punctate structures that form a ring-like structure after wortmannin treatment (Jaillais et al. 2007; Munch et al. 2015), we have a good reason to place the retromer core on the MVB membrane. Furthermore, the Rab5 GTPase RabG3f which is required for core retromer membrane attachment (Zelazny et al. 2013) also locates to a mature MVB, late prevacuolar compartment (Cui et al. 2014). In contrast, the SNXs are to be found at the TGN and maturing TGN elements (Ivanov et al. 2014; Niemes et al. 2010b; Robinson et al. 2012; Stierhof et al. 2013). These observations are supported by proteomic analysis of the endomembrane system (Heard et al. 2015), and suggest the presence of the two SNX subcomplexes in sequential compartments on the vacuolar pathway. One should note that the two have an overlap in localization in a subset of these compartments, as they have both been observed as punctate structures that form a ring-like structure after wortmannin treatment (Jaillais et al. 2006, 2007; Munch et al. 2015). Thirdly, there is now considerable evidence for plant SNXs participating in retromer-independent endomembrane-plasma membrane trafficking events (summarized in Heucken and Ivanov (2018)).

The belief that the retromer core is responsible for recycling VSRs is also not compatible with recently published data in mammalian systems. It turns out that it is the SNX heterodimers (SNX1 or SNX2 together with either SNX5 or SNX6) that bind to the recycling sorting motif WLM in the tail of the CI-MPR (Kvainickas et al. 2017; Simonetti et al. 2017). Accordingly, SNX knockouts caused an endosomal retention of CI-MPR whereas Vps35 knockouts did not. Interestingly, when SNX-BAR mutants are transiently expressed in tobacco leaf protoplasts already expressing fluorescently tagged VSRs, the steady-state distribution of the VSRs shifted towards the TGN (Niemes et al. 2010b). In contrast, under these conditions, de novo synthesized VSRs became trapped in the ER (Niemes et al. 2010a). These data suggest that it is again the SNXs rather than retromer, as originally thought, that mediate the trafficking of VSRs.

Another twist to the story is the nanobody technology–based data of Pimpl and coworkers (Fruholz et al. 2018; Kunzl et al. 2016) that has caused us to revise our thoughts about how and where VSR-ligand interactions take place in the secretory pathway. Contrary to the previously held dogma that VSRs cycle between MVBs and TGN, VSRs are now considered to recognize and interact with their cargo ligands in the ER, travel downstream to the TGN where the ligands dissociate. Ligand-free VSRs are then recycled back to the *cis*-Golgi for another round of anterograde transport. How and where then does retromer fit into this scenario, when its principal location is the MVB?

In the light of these facts, we would like to stress that caution needs to be exercised when discussing retromer in a plant context. We propose that proteins participating in the core VPS35-VPS29-VPS26 trimer should be labelled as “core retromer subunits” and the trimer as the “core retromer complex.” The SNX dimer should be treated as a separate complex. The general term retromer, suggesting a complex containing SNX proteins and the VPS35-VPS29-VPS26 trimer, should be avoided unless data is presented for its existence and functional significance. Due to the partly different subcellular localization patterns and functions of the SNX dimer and the VPS35-VPS29-VPS26 trimer, the term “retromer vesicles” should be avoided.

While the suggestion of Delgadillo et al. (2020) that EMACs may be functionally equivalent to the stationary compartments corresponding to the perinuclear TGN in mammalian cells is very speculative, the notion of a core retromer involvement in protein recycling to EMACs is worth discussing. That EMACs may be the place where recycled VSRs are delivered to for interaction with soluble intraluminal cargo is an attractive scenario. However, as discussed above, there is a discrepancy between retromer localization (MVBs) and the site of VSR-ligand dissociation, which now seems to be the TGN rather than MVBs. In addition, the possibility that SNXs are the binding partner for VSRs rather than retromer needs to be examined. In short, the roles of core retromer, the SNXs in the trafficking of VSRs, need to be carefully examined. We also need to know whether VSRs are present at EMACs, what portion of the ER-to-Golgi protein transport occurs through these compartments, and whether these soluble proteins are VSR cargo also needs to be addressed. Other open questions include in which way these compartments are associated with the ER, whether the Golgi apparatus or ER is their downstream compartment and which additional regulatory proteins/complexes are involved in EMAC function. Once VPS51 and/or VPS54 antibodies become available, it will be of great importance to obtain a description of these compartments at the ultrastructural level. Obtaining understanding in this direction might ultimately shed light on whether and how the core retromer and SNXs fit into this picture.

There is thus a clear contradiction between the site of retromer localization (MVBs) and the site from which VSRs are recycled (the TGN). After the submission of this manuscript, we became aware of the recent opinions article of Scheuring and Kleine-Vehn (2020) who also discussed the results of the Delgadillo paper. However, although they correctly give the TGN as the site from where VSRs are recycled, they place retromer binding and the formation of retrograde VSR carrying vesicles at the TGN (Fig. 1b). No mention of the sorting nexins is given. In Fig. 1c, we present a new model for the role of sorting nexins in VSR recycling. In comparison, the three main models are portrayed side by side.

## Data Availability

Not applicable.
